# Immune response to COVID‐19 vaccination is attenuated by poor disease control and antimyeloma therapy with vaccine driven divergent T‐cell response

**DOI:** 10.1111/bjh.18066

**Published:** 2022-02-17

**Authors:** Karthik Ramasamy, Ross Sadler, Sally Jeans, Paul Weeden, Sherin Varghese, Alison Turner, Jemma Larham, Nathanael Gray, Oluremi Carty, Joe Barrett, Stella Bowcock, Udo Oppermann, Gordon Cook, Chara Kyriakou, Mark Drayson, Supratik Basu, Sally Moore, Sarah McDonald, Sarah Gooding, Muhammad K. Javaid

**Affiliations:** ^1^ Oxford University Hospitals NHS Trust Radcliffe Department of Medicine Oxford University Oxford UK; ^2^ Department of Immunology Churchill Hospital, Oxford University Hospitals NHS, Foundation Trust Oxford UK; ^3^ The Botnar Research Centre Headington UK; ^4^ Late Phase Haematology Oxford University Hospital NHS Foundation Trust, Churchill Hospital Oxford UK; ^5^ Nuffield Department of Orthopaedics, Rheumatology and Musculoskeletal Sciences University of Oxford Oxford UK; ^6^ Oxford University Hospitals NHS Trust Oxford UK; ^7^ Princess Royal University Hospital, King's College Hospital Foundation NHS Trust Kent UK; ^8^ NIHR Leeds Medtech & In vitro Diagnostics Cooperative Leeds Teaching Hospitals Trust Leeds UK; ^9^ Department of Haematology University College London Hospitals NHS Trust London UK; ^10^ College of Medical and Dental Sciences Medical School University of Birmingham Birmingham UK; ^11^ University of Wolverhampton The Royal Wolverhampton NHS Trust Wolverhampton UK; ^12^ Oxford University Hospitals NHS Trust NHS Foundation Trust Oxford and Bath Royal United Hospitals Bath UK; ^13^ Myeloma UK Beaverbank Business Park Edinburgh UK; ^14^ MRC Molecular Haematology Unit, Weatherall Institute of Molecular Medicine University of Oxford Oxford UK; ^15^ Department of Haematology Oxford University Hospitals NHS Trust Oxford UK

## Abstract

Myeloma patients frequently respond poorly to bacterial and viral vaccination. A few studies have reported poor humoral immune responses in myeloma patients to COVID‐19 vaccination. Using a prospective study of myeloma patients in the UK Rudy study cohort, we assessed humoral and interferon gamma release assay (IGRA) cellular immune responses to COVID‐19 vaccination post second COVID‐19 vaccine administration. We report data from 214 adults with myeloma (*n* = 204) or smouldering myeloma (*n* = 10) who provided blood samples at least three weeks after second vaccine dose. Positive Anti‐spike antibody levels (> 50 iu/ml) were detected in 189/203 (92.7%), positive IGRA responses were seen in 97/158 (61.4%) myeloma patients. Only 10/158 (6.3%) patients were identified to have both a negative IGRA and negative anti‐spike protein antibody response. In all, 95/158 (60.1%) patients produced positive results for both anti‐spike protein serology and IGRA. After adjusting for disease severity and myeloma therapy, poor humoral immune response was predicted by male gender. Predictors of poor IGRA included anti‐CD38/anti‐BCMA (B‐cell maturation antigen) therapy and Pfizer‐BioNTech vaccination. Further work is required to understand the clinical significance of divergent cellular response to vaccination.

## INTRODUCTION

High mortality rates with COVID‐19 in myeloma patients coupled with anticipated poor COVID‐19 vaccine responses will increase isolation periods and be detrimental to their myeloma care.^1^ Myeloma patients have been shielding since the start of the COVID‐19 pandemic. This has resulted in disruption to therapy and healthcare provision, significant social isolation and worse mental health.^2^, ^3^ The solution to this is providing meaningful protection against COVID‐19 through vaccination and ensuring this protection is durable.

Myeloma is a malignant clonal proliferation of post germinal‐centre B cells and plasma cells. The canonical role of these immune cells is to provide both immunity against pathogens, respond to vaccination and deal with pathogens in mucosal surfaces with IgA production. Prolonged SARS‐CoV‐2 viral shedding and delayed or negligible seroconversion has been observed in infected patients.^4^ Myeloma commonly affects the elderly population, a cohort that principally suffers with co‐morbidities and immunosenescence, further increasing patients’ risk of developing complications from infections and poor response to vaccination. Myeloma as a disease requires ongoing immune suppressive chemotherapy to induce and maintain remission which results in frequent medical visits and exposure to hospital environment. Influenza vaccination studies report that myeloma patients often require repeated doses of vaccine to mount an optimal antibody response.^5^ Pneumococcal vaccination response in myeloma is frequently impaired and was found to be associated with poor disease control in a study of myeloma patients given 23‐valent polysaccharide vaccine.^6^ The poor seroconversion that results from established cellular and humoral immune dysfunction can be exacerbated by active treatment and disease status.^7^ Humoral immune response to COVID‐19 vaccination in myeloma patients given two doses of mRNA‐based COVID‐19 vaccination ranged between 80% and 85% in three separate cohort studies.^8^, ^9^, ^10^ However, the protective titre of antibodies required to prevent re‐infection (neutralisation) is unclear, as is the ability to protect patients from other SARS‐CoV‐2 virus variants of concern (VOC). Moreover, the role of the cellular immune response to SARS‐CoV‐2 is crucial, and to date has not been assessed in a large cohort of multiple myeloma (MM) patients.

In the UK both mRNA‐based (Pfizer‐BioNTech; PB) and viral vector‐based vaccination (Oxford/Astra Zeneca; AZ) were offered to patients. Also, the recommended schedule of 12 weeks for second dose was divergent from manufacturer's recommendations. It is unclear whether these play a role in vaccination responses in myeloma patients. Understanding drivers of poor response to vaccination and potential salvage strategies (i.e. booster, passive antibody) for this subset is key to managing their myeloma optimally.

In order to address these evidence gaps, all of which directly affect patient management decisions, both immediately and booster dose planning, we initiated a national web‐based prospective study of adults with MM to determine the humoral and cellular responses post the completion of the first and second dose of COVID‐19 vaccine schedule administered in the UK in 2021.

## METHODS

This is a prospective, observational study. The study is based on the existing RUDYstudy.org platform (LREC 14/SC/0126, RUDY LREC 17/SC/0501), an established online rare‐disease platform with online dynamic consent and patient‐reported data.^11^ South Central ‐ Berkshire B Research Ethics Committee approved the study.

### Recruitment

To ensure reaching our recruitment target rapidly, multiple pathways for recruitment were employed. The study is currently open to any UK resident to join via 30 hospitals and through the Myeloma UK national patient charity and Blood Cancer UK digital platform. Informed online dynamic consent was obtained from all participants.

### Assessment

**FIGURE 1:**
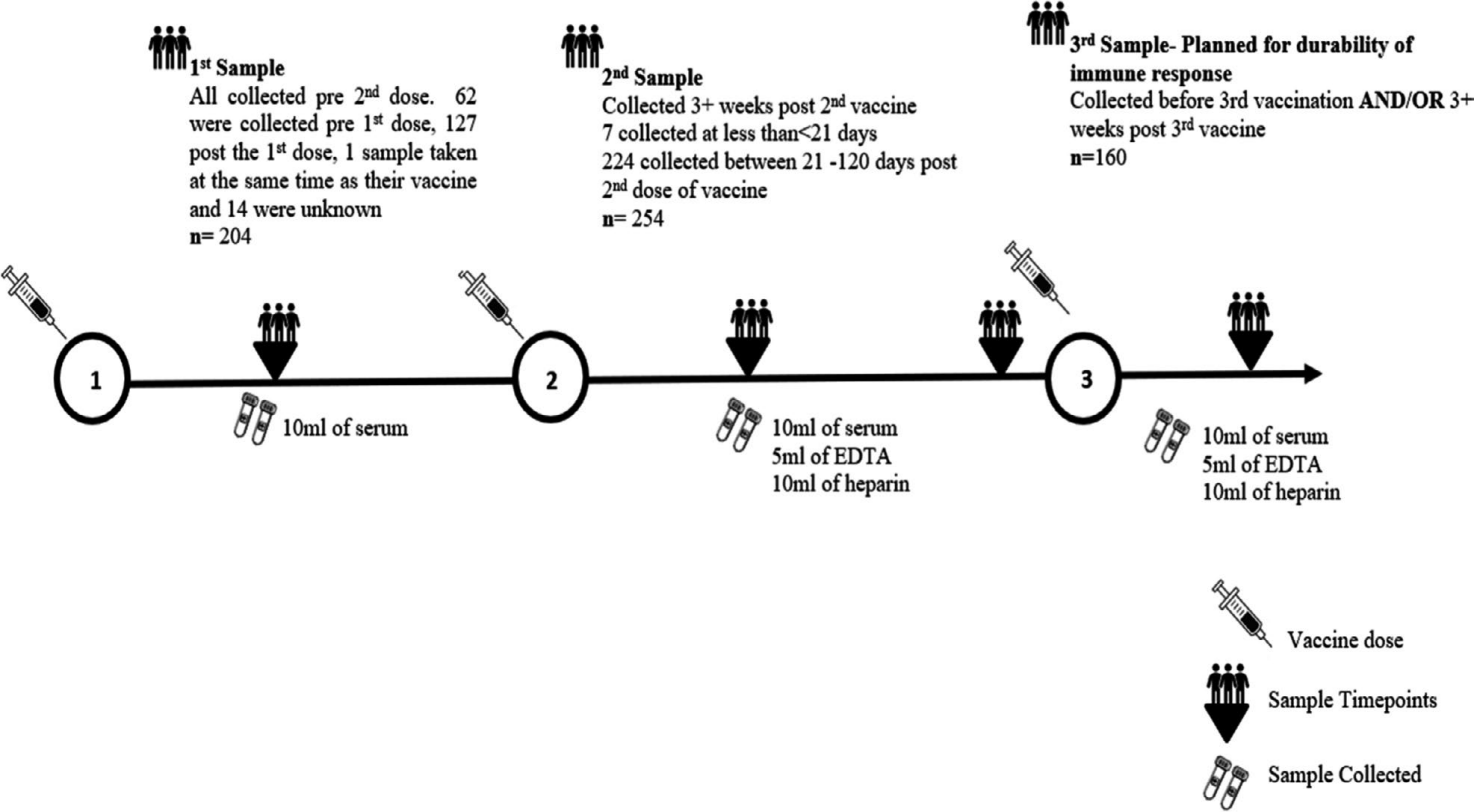
Blood sampling plan aligned to vaccination with sample numbers and type of samples obtained [Colour figure can be viewed at wileyonlinelibrary.com]

Participants were able to enter online the time of their first and second vaccine doses. Based on these dates, a sample box was posted to and received from the participants, containing serum, EDTA and heparin blood tubes after the second vaccine dosing (Figure 1).

In addition, participants completed online questionnaires that included COVID‐19 symptoms, testing and myeloma features such as current myeloma status (see Supplementary File 1). Independently, there was review of clinical data to identify myeloma status and treatment at the time of second vaccine dose in a subset of participants. If the clinical data were not available for myeloma status, the closest patient‐reported myeloma status was used.

### Laboratory assessments

Collected serum samples were tested for antibodies against SARS‐CoV‐2 nucleocapsid (N) or spike (S) protein. SARS‐CoV‐2 N protein antibodies were measured by turbidimetry (Abbott), with samples that produced values of >1.4 iu/ml considered to be positive. SARS‐CoV‐2 S protein antibodies were measured by turbidimetry (Abbott) (IgG serology only), with a cut‐off value of 50 iu/ml considered to be a positive result. Heparin samples collected from patients were used to isolate peripheral blood mononuclear cells (PBMCs), which were then placed into a SARS‐CoV‐2 S protein interferon gamma release assay (IGRA) (Oxford immunotec T IGRA) to quantify SARS‐CoV‐2‐specific effector T cells. The assay was followed as per the kit insert with positive results defined as >8 interferon gamma‐releasing cells/10^6^ PBMCs. Total lymphocyte count was determined by flow rate analysis, as part of a lymphocyte subset panel (Beckmann Coulter). A concentration of <1.5 × 10^9^ was used to classify lymphopenia. Total IgG levels were measured by turbidimetry (Abbott).

### Statistical analysis

Descriptive statistics included *t* tests and ANOVA for parametric outcome measures, Spearman correlations, and Kruskall–Wallis tests for non‐parametric outcomes. Categorical results were evaluated by Chi‐squared and Fisher's exact tests when individual cell counts were less than 10. Multivariate logistic regression analyses were performed to identify independent predictors of anti‐S antibody response and IGRA positivity. We combined humoral and T‐cell outcomes responses to generate four independent groups: combined positive anti‐S antibody and IGRA reactivity compared with those with either anti‐S antibody or IGRA reactivity and those with double‐negative results. These groups were analysed with multinomial regression. Significance was determined as *p* < 0.05.

## RESULTS

Two hundred and fourteen participants with myeloma (*n* = 204) or smouldering myeloma (*n* = 10) completed the COVID‐19 questionnaire and returned a blood sample at least three weeks after their second dose of COVID‐19 vaccine (median 9.5 weeks (range 3–20.4 weeks). The baseline characteristics are shown in Table 1, with over 30% of participants aged 70 years and over. The type of vaccine used was reported by 160 participants with the AZ vaccine reported by 59.6% of patients with myeloma and 44.4% of patients with smouldering myeloma and the PB vaccine used by the remainder. Neither age, sex, myeloma status or chemotherapy at time of vaccination predicted the type of vaccine used (*p* > 0.1). However, the PB vaccine was given an average of six days before AZ vaccine (*p* = 0.01).

**TABLE 1:** Baseline characteristics of patients included in the analysis of immune response post vaccination

Characteristics		All participants	Myeloma	Smouldering myeloma
		*n* = 214	*n* = 204	*n* = 10
Female (%)		94 (43.5%)	88 (43.1%)	6 (60%)
Age (SD)		64.8 (9.1)	64.9 (9.1)	63.8 (8.3)
Age ≥ 70 years (%)		70 (32.7%)	67 (32.8%)	3 (30.0%)
Ethnicity	White UK (%)	188 (87.9%)	181 (88.7%)	7 (70.0%)
Myeloma status^a^ (%)	CR/ VGPR		102 (50.0%)	
	PR/stable		50 (24.5%)	
	Progression/relapse		34 (16.7%)	
	Unknown		18 (8.8%)	
Type of vaccine	Oxford/AstraZeneca	94 (43.9%)	90 (44.1%)	4 (40%)
	Pfizer‐BioNTech	66 (30.8%)	61 (29.9%)	5 (50%)
	Unknown	54 (25.2%)	53 (26.0%)	1 (10%)
Chemotherapy (*n* = 108)	No therapy		40 (19.6%)	
	CD38 antibody or BCMA		32 (15.7%)	
	Other		49 (24.0%)	
	Unknown		83 (40.7%)	
COVID‐19 reported characteristics	Major symptoms	8	8	0
	History of testing	127	121	6
	Positive test result	2	2	0

Abbreviations: CR/VGPR, Complete remission/very good partial remission; PR/stable, partial remission/stable disease.

^a^ At time of second vaccine dose.

The humoral response findings after the first and second COVID‐19 vaccine dose are shown in Table 2, with the distribution of individual antibody results for all participants shown in Figure S1. Serological evidence of prior COVID‐19 infection, by the detection of anti‐N antibodies, after the second vaccine dose was found in seven participants, six with a diagnosis of myeloma and one with a diagnosis of smouldering myeloma. No individual acquired anti‐N‐positive status between the first and second vaccine dose. Those with a positive anti‐N antibody (natural infection) at second sample had a significantly higher anti‐S protein response (*p* = 0.002) (Figure S2).

**TABLE 2:** COVID‐19 S and N protein antibody status after first and second vaccine dosing

	All participants	Myeloma	Smouldering myeloma
Post 1st vaccine dose	*n* = 154	*n* = 146	*n* = 8
S protein antibody ≥50 iu/ml^a^	50 (67.6%)^a^	46 (66.7%)^a^	4 (80%)^a^
N protein antibody≥1.5 iu/ml	5 (3.3%)	5 (3.4%)	0
Post 2nd vaccine dose	*n* = 214	*n* = 203	*n* = 10
S protein antibody ≥50 iu/ml	198 (92.5%)	189 (92.7%)	9 (90.0%)
N protein antibody ≥1.5 iu/ml	7 (3.3%)	6 (3.0%)	1 (9.1%)

^a^ Restricted to 73 participants (69 myeloma and 4 smouldering myeloma) whose sample was at least 21 days after the first dose.

**FIGURE 2:**
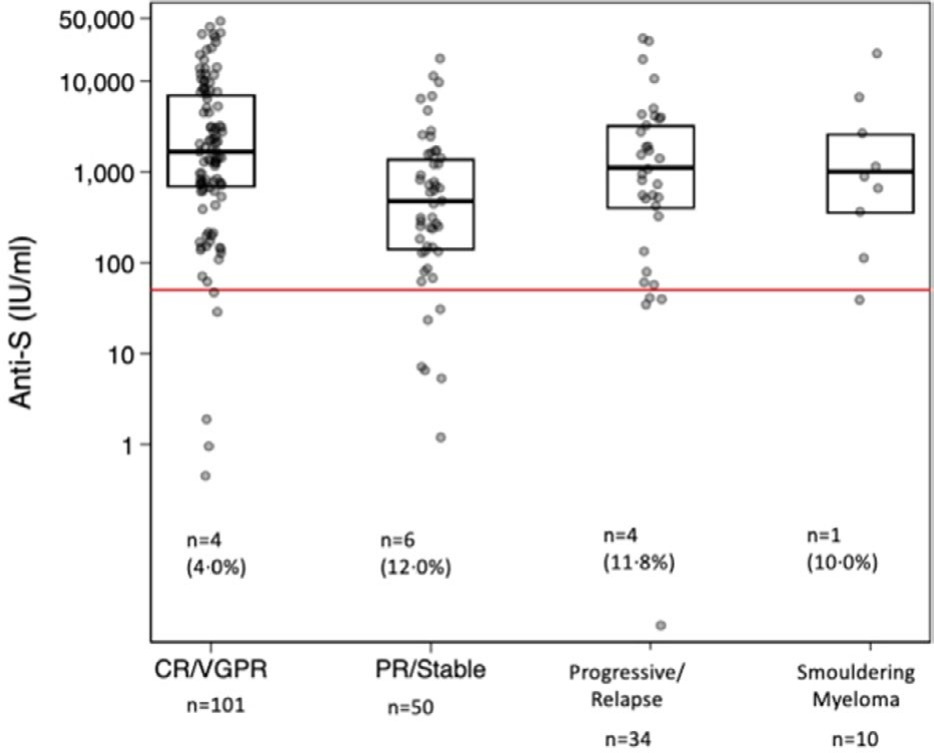
Relationship between anti‐S antibody concentration after second vaccine dose and myeloma status from clinical records and patient reported (*n* = 195) CR/VGPR, Complete remission/very good partial remission; PR/stable, partial remission/stable disease. Kruskal–Wallis test, *p* < 0.001 for myeloma patients only (excluding smouldering myeloma patients) [Colour figure can be viewed at wileyonlinelibrary.com]

The comparison between S protein antibody levels after the first and second vaccine are shown in Figure S3. One participant had sufficient anti‐S antibody after the first vaccine dose (anti‐S 1268 iu/ml) but not after the second dose (anti‐S 41.2 iu/ml). This patient had smouldering myeloma and received a dose of rituximab for an inflammatory arthritis between vaccine doses. Anti‐S levels by myeloma status and in smouldering myeloma are shown in Figure 2. A low anti‐S concentration was more common in patients with partial response/stable disease or progressing/relapsing disease compared with those in complete remission/very good partial remission (11.9% vs 3.9%, *p* = 0.051). Men with myeloma were significantly more likely to have low anti‐S levels (<50 iu/ml) after second vaccination compared with women (11.3% vs 2.3%, *p* = 0.015), with no difference by age (*p* = 0.46). Men were more (*p* = 0.028) likely to have a low anti‐S level after adjusting for serum IgG levels and this was borderline significant (*p* = 0.07) after adjusting for total lymphocyte count. Anti‐S levels were overall negatively associated with age (Figure S4A). Participants who reported receiving the PB *versus* AZ vaccine were no different in achieving a satisfactory anti‐S concentration (89.4% vs 93.6% respectively) but had higher anti‐S antibody concentrations (*p* = 0.018) (Figure S4B). The time difference between the second vaccine dose and sample collection date was negatively correlated with anti‐S concentration (Spearman rho = −0.21, *p* = 0.002) but not with anti‐S concentrations above or below 50 iu/ml (*p* = 0.78) (Figure S5A). The median interval between the first and second vaccine dose was 11 weeks (range 2 to 12.7 weeks) and was not associated with anti‐S concentration after the second vaccine dose (*p* = 0.22) (Figure S5B).

**FIGURE 3:**
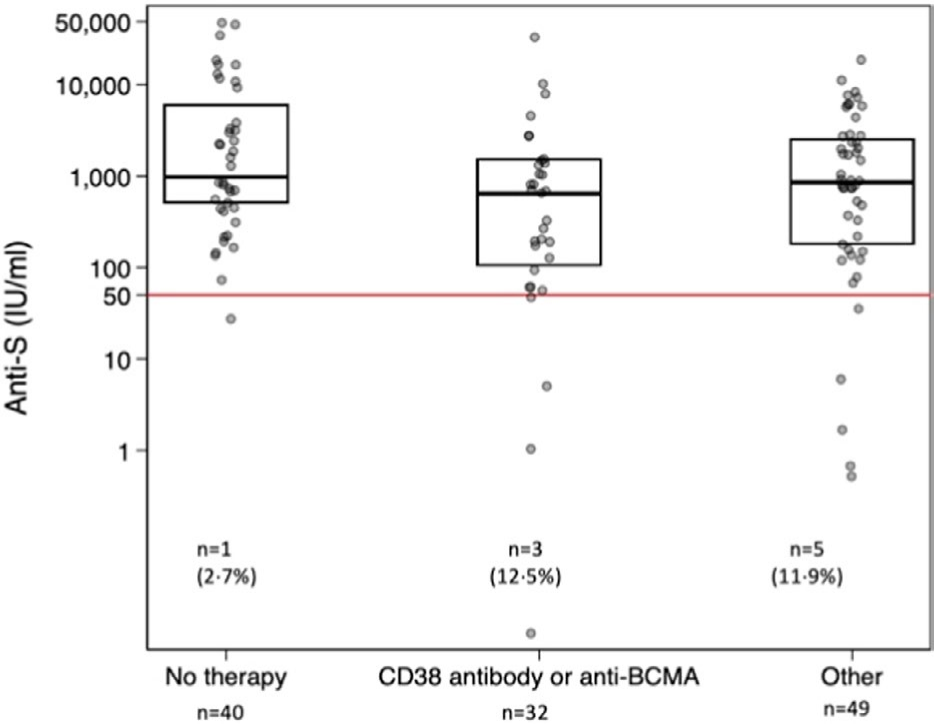
Relationship between anti‐S antibody concentration and chemotherapy at time of second vaccine dose (*n* = 121) CD38 antibody—daratumumab, isatuximab; BCMA ADC (B‐cell maturation antigen antibody–drug conjugate)—belantamab. Other: proteasome inhibitors—ixazomib, carfilzomib, bortezomib; immunomodulatory drugs—thalidomide, lenalidomide, pomalidomide, bendamustine, cyclophosphamide, dexamethasone, other steroids. Kruskal–Wallis for anti‐S level, *p* = 0.027. Fisher exact test for anti‐S <> 50 iu/ml, *p* = 0.26

Chemotherapy data were available for 126 patients at time of second vaccination from their clinical records. Of the myeloma patients, 45 were on no treatment. Thirty‐two patients were on CD38 antibody or BCMA‐containing regimens. Other non‐CD38 regimens included proteasome inhibitors, immunomodulatory, alkylating chemotherapy and steroids (*n* = 49). Those on chemotherapy had lower levels of anti‐S than those not on chemotherapy (*p* = 0.025) (Figure 3). Using a threshold of 50 iu/ml to identify participants with suboptimal levels, the proportion of myeloma patients with <50 iu/ml of anti‐S was non‐significantly higher in those on chemotherapy (anti‐CD38/anti‐BCMA, three; other chemo, five) *versus* those not on chemotherapy [eight (9.6%) *versus* one (2.2%), *p* = 0.16].

COVID‐19 IGRA was measured after the second dose of vaccine, with results in 167/214 participants (Table 3). Positive IGRA results were significantly (*p* = 0.002) associated with anti‐S serology status after second vaccine. Participants were more likely to be IGRA‐negative if they were not in CR/VGPR (*p* = 0.021) with no significant differences by chemotherapy status (Table S1). Participants receiving the AZ vaccine had a significantly higher IGRA reactivity rate, 70.6%, than those who received the PB, 44.2% in all participants, and those with myeloma (Table 4).

**TABLE 3:** Relationship between S protein antibody status and T‐cell response in all participants after the second COVID‐19 vaccine dose

		Anti‐S status after second vaccine					
		All participants		Myeloma		Smouldering myeloma	
		<50 iu/ml	≥50 iu/ml	<50 iu/ml	≥50 iu/ml	<50 iu/ml	≥50 iu/ml
T spot		*n* = 12	*n* = 155	*n* = 12	*n* = 146		*n* = 9
Negative	*n* = 65	10 (15.4%)	55 (84.6%)	10 (16.4%)	51 (83.6%)		4
Positive	*n* = 102	2 (2.0%)	100 (98.0%)	2 (2.1%)	95 (97.4%)		5

*n* = 167.3 patients had indeterminate T‐spot results whose data not shown. Row percentages shown. Fisher's exact *p* = 0.001 for all participants and myeloma participants.

**TABLE 4:** Relationship between IGRA reactivity and type of COVID‐19 vaccine

	All participants (*n* = 119, *p* = 0.002^a^)		Myeloma patients (*n* = 111, *p* = 0.006^a^)		Smouldering myeloma (*n* = 8, *p* = 0.20^b^)	
T spot	Oxford/AstraZeneca	Pfizer‐BioNTech	Oxford/AstraZeneca	Pfizer‐BioNTech	Oxford/AstraZeneca	Pfizer‐BioNTech
Negative	20 (28.2%)	27 (56.3%)	20 (29.4%)	24 (55.8%)	0 (0%)	3 (60%)
Positive	51 (71.8%)	21 (43.8%)	48 (70.6%)	19 (44.2%)	3 (100%)	2 (40%)

Abbreviation: IGRA, interferon gamma release assay.

^a^ Chi‐squared test.

^b^ Fisher exact test.

The multivariate models for determinants of an anti‐S concentration of less than 50 iu/ml are shown in Table 5. Women were significantly less likely to have a low anti‐S concentration (<50 iu/ml) than men, even after adjusting for myeloma status. For IGRA reactivity, increased age but not gender predicted a negative result, as did CD38 antibody/anti‐BCMA Ab exposure and receiving PB vaccine (Table 6). The IGRA reactivity rate in participants who received the AZ vaccine remained significant after adjusting for age, sex and myeloma status (*p* = 0.006) and borderline significant after adjusting for chemotherapy (*p* = 0.051). When analysing the different combinations of anti‐S and IGRA status, progressive disease/relapse myeloma status predicted double‐negative status [relative risk ratio (RRR) 9.6, 95% confidence interval (CI) 1.44–63.07] after adjusting for age and sex. Having a positive anti‐S level with a negative IGRA release assay was less common (*p* = 0.004) with the AZ than PB vaccine (RRR 0.23, (95% CI 0.09–0.63)) after adjusting for age, sex and myeloma status.

**TABLE 5:** Independent predictors of low anti‐S concentration (≤50 iu/ml) post second vaccination in myeloma patients only

Predictor	Model 1 (*n* = 203)		Model 2 (*n* = 186)		Model 3 (*n* = 120)	
	Odds ratio	95% CI	Odds ratio	95% CI	Odds ratio	95% CI
Age (year)	1.04	0.97–1.12	1.01	0.95–1.09	1.06	0.96–1.18
Female sex	**0.21**	**0.05–0.96**	**0.21**	**0.04–1.00**	0.22	0.039–1.29
Time between vaccine doses (weeks)	0.8	0.63–1.13				
Time from second vaccine dose and sample (weeks)	0.92	0.78–1.08				
Myeloma status ‐partial response/stable^a^			3.37	0.86–13.24	2.37	0.50–11.19
Myeloma status ‐progression/relapse^a^			2.81	0.62–12.76	3.27	0.26–40.55
CD38/BCMA^b^					5.03	0.63–56.42
Other chemo^b^					5.98	0.63–56.42
Pseudo‐R^2^	0.09		0.10		0.15	

^a^ Complete remission/very good partial response is comparator.

^b^ No chemotherapy is comparator.

Bold indicates statistically significant.

**TABLE 6:** Independent predictors of negative T‐spot post second vaccination in myeloma patients only

Predictor	Model 1 (*n* = 157)		Model 2 (*n* = 105)		Model 3 (*n* = 68)	
	Odds ratio	95% CI	Odds ratio	95% CI	Odds ratio	95% CI
Age (year)	**1.04**	**1.01–1.09**	1.03	0.98–1.09	1.04	0.97–1.11
Female sex	0.84	0.43–1.64	1.05	0.43–2.60	1.06	0.32–3.77
Time between vaccine doses (weeks)	1.02	0.85–1.64				
Time from second vaccine dose and sample (weeks)	0.98	0.89–1.07				
Oxford/AstraZeneca vacccine^a^			**0.27**	**0.11–0.69**	0.29	0.08–1.01
Myeloma status ‐partial response/stable^b^			**2.88**	**1.04–7.97**	2.41	0.67–8.73
Myeloma status ‐progression/relapse^b^			2.30	0.76–6.96	0.93	0.13–6.52
CD38/BCMA^c^					**4.80**	**1.09–21.02**
Other chemo^c^					1.21	0.31–4.70
Pseudo‐R^2^	0.08		0.12		0.15	

^a^ Pfizer‐BioNTech is comparator.

^b^ Complete remission/very good partial response is comparator.

^c^ No chemotherapy is comparator.

Bold indicates statistically significant.

## DISCUSSION

Our prospective study has shown that following two doses of COVID‐19 vaccination, myeloma patients can elicit anti‐S protein antibodies in a significant proportion (92.7%) of patients. Despite its virtual consenting platform and requirement of IT literacy, it had recruited 32.7% of patients aged 70 and over, with representative ethnicity, and therefore results can be extrapolated to the wider UK population. T‐cell responses also contribute to immunity and only a proportion of patients have elicited T‐cell responses after two doses. T‐cell responses following vaccination are not routinely studied and it is less clear how this would affect ability of patients to neutralise SAR‐CoV‐2 infection. But a very small proportion of myeloma patients, 10/158 (6.3%), lacked both humoral and T‐cell response to vaccination. These patients should be directed to salvage strategies such as further doses of vaccination and passive antibody therapy to prevent severe COVID‐19 (Figure S6). Patients showed a significant uplift of antibody response to second vaccination dose in 31% of patients (Figure S2). There are ongoing trials of passive antibody treatment for patients with poor antibody response.^12^

Patients with smouldering myeloma elicited a robust anti‐S antibody response to one dose of the vaccine, with one patient losing antibody response following rituximab therapy for concomitant arthritis. This highlights that the durability of immune response can be affected by immunosuppressive agents. Prior COVID‐19 infection significantly improved response to COVID‐19 vaccination, which has also been observed in other haematological oncology patients.^8^ One concern was the ability of AZ vaccine, which is viral vector‐based, in eliciting immune responses in myeloma patients. We found two doses of AZ vaccine gave a similar high proportion of patients a satisfactory humoral response using a threshold of >50 iu/ml as the Pfizer‐BioNTech vaccine, but with a higher T‐cell response in myeloma patients. Previously, following one dose of PB *versus* AZ vaccination in 165 elderly immunosenescent population (80 year or older) significantly better T‐cell responses were noted after one dose of AZ vaccination. Our study extends this observation to two doses. The clinical implication of this is currently unclear and whether AZ vaccination induces better durability of both humoral and T‐cell response requires further follow‐up data.^13^ Despite there being a 12‐week dosing interval imposed in the UK we were not able to demonstrate a significant difference in anti‐S responses between patients with increasing intervals between the first and second vaccine dose. Samples were obtained at varying timepoints in patients following the second dose of vaccine, and a significant difference was seen in relation to time of sample post vaccine, with falling anti‐S antibody response with time, suggesting decay. This requires further follow‐up and could be mitigated by planned third primary dose vaccination six months post second dose for myeloma patients in the UK. We have planned further sampling to assess durability of immune response prior to third dose (Figure 1). Both T‐cell and anti‐S antibody response were dampened in patients who had partial response/stable disease or had relapsing/progressing disease. Poor disease control is a predictor of double‐negative (T‐cell and humoral) immune response. This suggests that robust disease control should be secured prior to vaccination, or vaccination prior to intensive treatment, particularly during future booster vaccination doses in patients. We found up to 12% of patients who were on therapy that had low titres, below threshold of anti‐S antibody in comparison with patients not on therapy, but this was not significant. There was a similar non‐significant trend noted in T‐cell response data analysis.

Previous studies have shown robust antibody responses in a comparable proportion of myeloma patients when measured on different antibody assay platforms to that reported here.^8^, ^9^, ^10^ Data from Ehsam *et al*. show up to 50% of myeloma patients elicited a T‐cell response.^10^ This showed a good correlation with anti‐S antibody response as observed in our patient population. Data from Terpos *et al*. who reported on neutralisation antibody responses showed only approximately 70% of myeloma patients have detectable levels.^14^ This may be a better functional assay to use but not available in routine practice. In a small cohort of myeloma patients Bitoun *et al*. showed a good correlation between titres of anti‐Spike IgG levels and neutralisation ability.^15^ If these data are confirmed in larger cohorts and neutralisation against VOC, a widespread use of humoral anti‐S antibody in routine practice can be considered. Both Terpos *et al*. and Van Oeklen *et al*. have shown data that ongoing therapy particularly with anti‐CD38 antibodies and BCMA‐targeted therapy significantly reduced antibody responses in myeloma patients.^9^, ^14^ We observed a higher proportion of patients on therapy having lower antibody levels, but we could not find a difference between the types of antimyeloma therapies administered. There may be differences in how these therapies have been applied (combinations), in doses and/or duration of therapy which may explain the variability observed.

Data from Terpos *et al*.^16^ and Greenberger *et al*.^17^ confirm our observation of better humoral responses to vaccination in women compared to men. It is currently unclear why a differential immune response is observed. But immune response to natural SARS‐CoV‐2 infection is also variable with a dominant adaptive immune response observed in women compared to men.^18^ In comparison with immune response data reported following vaccination in lymphoma and chronic lymphocytic leukaemia (CLL) patients, myeloma patients are able to elicit better humoral and T‐cell responses.^8^, ^10^ But a small proportion of patients (6%) are antibody‐ and IGRA‐negative and require salvage strategies to mitigate against infection. Future work is needed to understand if more detailed immunophenotyping predicts immune response to COVID‐19 vaccination.

### Strengths and weaknesses of the study

The strength of the study is confirmation of an immune response in heterogeneously vaccinated patients with longer than recommended duration between first and second dose. Our humoral response results are comparable to data reported so far. We have generated a large dataset of T‐cell response alongside humoral response in myeloma patients. Our data have also been generated in patients with a comparable demography to the UK myeloma population. Studies use different platforms to generate both antibody and T‐cell results which makes direct comparability difficult. Our study is limited by the missing data on chemotherapy and myeloma status from the clinical notes for all participants. We partially addressed this by adding self‐reported myeloma status where clinical data were not required, and when we compared using only clinical record data the findings were similar.

Further follow‐up to determine whether immune response wanes over time and whether use of particular therapies has a more significant detrimental effect on vaccine response requires evaluation. A subset of patients who require alternative strategies to prevent COVID‐19 such as passive antibody therapy^12^ or prophylactic antiviral therapy should be studied in prospective trials in this patient population.^19^ Whilst these laboratory results are reassuring, the clinical implication of the observed humoral/cellular immune responses and real‐world infection rates and severity of infection remains unanswered and highlights the complexity of the immune responses to different COVID‐19 vaccines.

In conclusion, a robust humoral (92.7%) anti‐S antibody response can be elicited following either AZ or Pfizer mRNA vaccination in myeloma patients. A 12‐week dosing interval is not detrimental to immune response. Encouragingly, a good proportion (60.1%) have also elicited T‐cell response. AZ vaccination provides robust T‐ cell responses in myeloma patients. Data on durability of immune responses and including the role of factors such as ongoing therapy and further vaccine doses require further follow‐up. Ongoing collection of data during patient follow‐up, including incidence of SARS‐CoV‐2 infection and severity of COVID‐19, would provide further clinical significance of the immune response elicited by vaccination, for myeloma patients and their carers.

## CONFLICT OF INTERESTS

All authors completed the ICMJE disclosure form. Authors does not disclose any direct conflict of interest in this submitted work. The following personal or financial relationships NOT RELEVENT to this manuscript existed during the conduct of the study. Mark Drayson reports shares in Abingdon Health. Sarah McDonald reports honoraria from Takeda, Janssen, Sanofi and Celgene. Sally Moore reports honoraria from Takeda, Janssen, Sanofi and Celgene. Gordon Cook reports advisory board from Takeda, Celgene, Janssen, Sanofi, Oncopeptide, Roche, Karyopharm, IQVIA and Amgen. He also reports research grants from Celgene, Takeda and IQVIA. Nathanael Gray reports honoraria from Janssen and Amgen and research grant from Kyowa Kirin. Supratik Basu reports advisory board from Pfizer and SANOFI. Muhammad Javaid reports research grant from Amgen. Karthik Ramasamy reports honoraria, research grant from Janssen, Celgene, Takeda and Amgen. He also reports advisory board from Celgene, Takeda, Janssen, Amgen, Abbvie, Sanofi, Oncopeptides, Karyopharm, GSK, Adaptive biotech, Pfizer and speaker's bureau from Celgene, Takeda and Adaptive Biotech.

## AUTHOR CONTRIBUTIONS

All listed authors made substantial contributions to the conception or design of the work; or the acquisition, analysis, or interpretation of data for the work; and drafting the work or revising it critically for important intellectual content; final approval of the version to be published; and agreement to be accountable for all aspects of the work in ensuring that questions related to the accuracy or integrity of any part of the work are appropriately investigated and resolved. Karthik Ramasamy is the guarantor and accepts full responsibility for the work and/or the conduct of the study, had access to the data, and controlled the decision to publish. The lead authors (Karthik Ramasamy, Ross Sadler and Muhammad K. Javaid) designed the study and wrote the report. All authors were study investigators. Sally Jeans and Paul Weeden were patient advisors. Ross Sadler, Sherin Varghese, Alison Turner, Jemma Larham and Oluremi Carty collated the data. Karthik Ramasamy, Ross Sadler and Muhammad K. Javaid analysed the data. All authors contributed to the review of the manuscript and approved the final version before submission.

## Funding information

Funding for this study has been received from Blood Cancer Vaccine Consortium and Janssen UK. RUDY platform has been funded by National Institute for Health Research.

## PUBLIC AND PATIENT INVOLVEMENT STATEMENT

RUDY patients forum, Oxford Blood Group, Myeloma UK patient research panel were all consulted, and feedback secured on study design, questionnaire.

## TRANSPARENCY DECLARATION

Karthik Ramasamy, the lead author (and the manuscript's guarantor), affirms that the manuscript is an honest, accurate, and transparent account of the study being reported; that no important aspects of the study have been omitted; and that any discrepancies from the study as planned (and, if relevant, registered) have been explained.

## Supporting information


Figure S1
Figure S2
Figure S3
Figure S4
Figure S5
Figure S6
Table S1

RUDY COVID‐19 questionnaire

## Data Availability

Patients have consented to anonymised data sharing for research and publication purposes.
