# Supplementary material for: Immune response to COVID‐19 vaccination is attenuated by poor disease control and antimyeloma therapy with vaccine driven divergent T‐cell response
Source: Br J Haematol. 2022 Feb 17;197(3):293–301. doi: 10.1111/bjh.18066 (PMC9306522; doi:10.1111/bjh.18066)
Supplement: Supplementary file 1 — Figure S1 Figure S2 Figure S3 Figure S4 Figure S5 Figure S6 Table S1 [file BJH-197-293-s002.pdf]

Supplementary Figures

Supplementary Figures and legends

Supplementary Figure 1	Post vaccination S and N protein antibody levels
Supplementary Figure 2	Relationship between Anti-S concentration after second vaccine dose and serological evidence of past COVID infection<br>*Legend: Kruskal Wallis test p<0.001*
Supplementary Figure 3	Relationship between Anti-S antibody concentration after 1st vs 2nd vaccine dose (n=74)
Supplementary Figure 4a	Relationship between Anti-S concentration after second vaccine dose and age<br>*Legend: Lowess line shown. Spearman rho -0.15, p=0.021*
Supplementary Figure 4b	Comparison of Anti-S antibody after second vaccine dose by type of Vaccine<br>*Legend: Comparison of Anti-S values < 50 IU/ml, using Fischer exact test, p=0.39 Kruskal-Wallis p=0.018*
Supplementary Figure 5a and 5b	Relationship between Anti-S concentration and a) time from second vaccine; b) interval between vaccine doses<br>*Legend: (a) Dash-lines represents Lowess line with 0.8 bandwidth, Solid red line, threshold of 50 IU/ml. Spearman's rho= -0.12, p=0.002. (b) Dash-lines represents Lowess line with 0.8 bandwidth, Solid red line, threshold of 50 IU/ml. Spearman's rho= 0.085, p=0.22*
Supplementary Figure 6	Visual representation of T-spot and humoral responses post vaccination

Supplementary Figure 1. Post vaccination S and N protein antibody levels

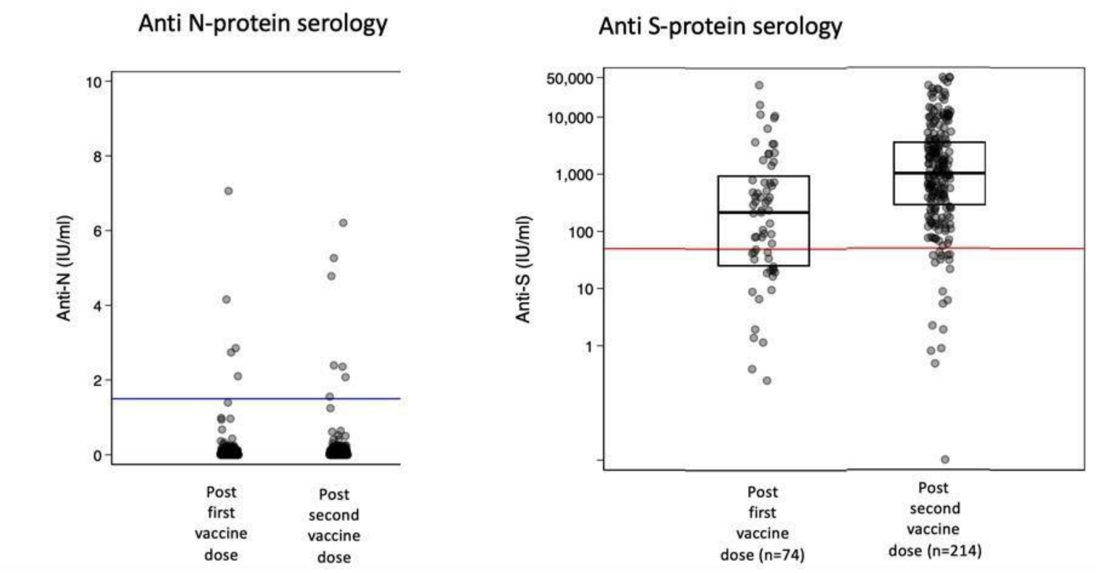

Supplementary Figure 2. Relationship between Anti-S concentration after second vaccine dose and serological evidence of past COVID infection

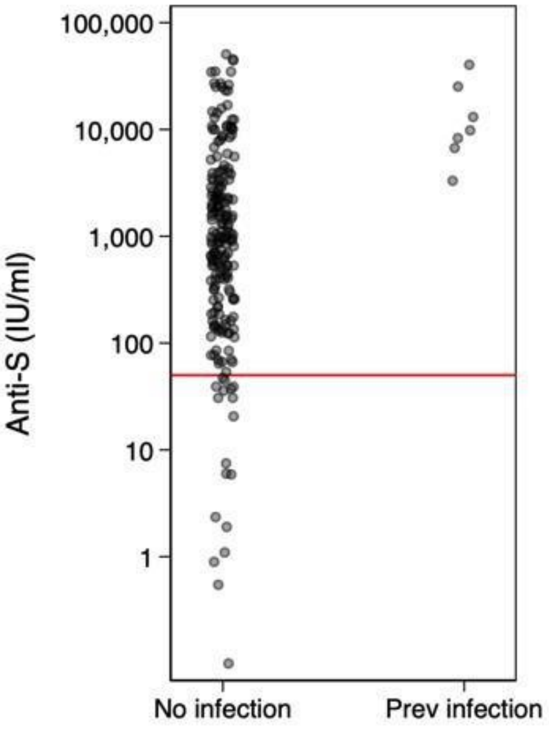

Legend: Kruskal Wallis test $p<0.001$l

Supplementary Figure 3. Relationship between Anti-S antibody concentration after 1st vs 2nd vaccine dose (n=74)

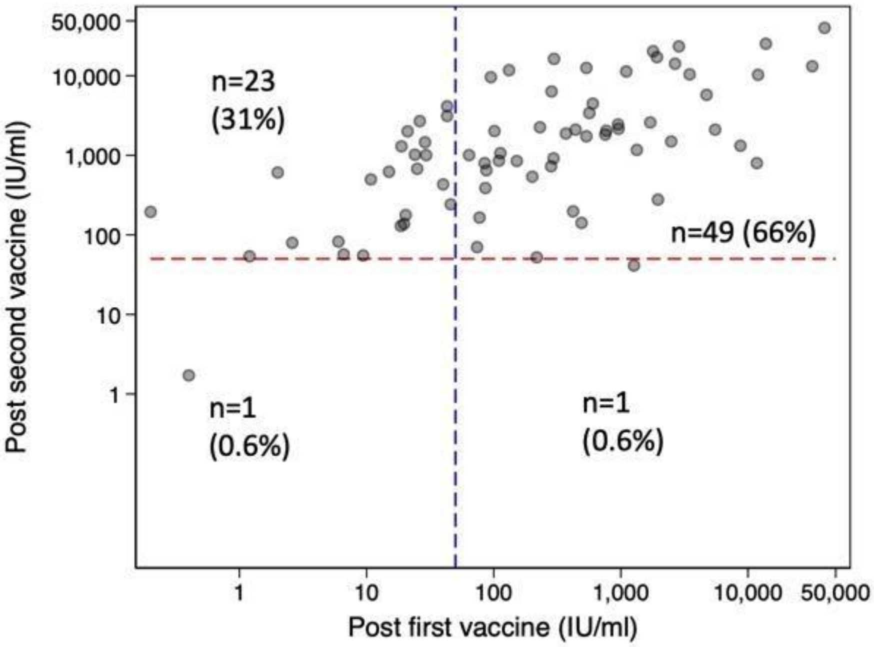

Supplementary Figure 4a. Relationship between Anti-S concentration after second vaccine dose and age

Legend: Lowess line shown. Spearman rho -0.15, p=0.021

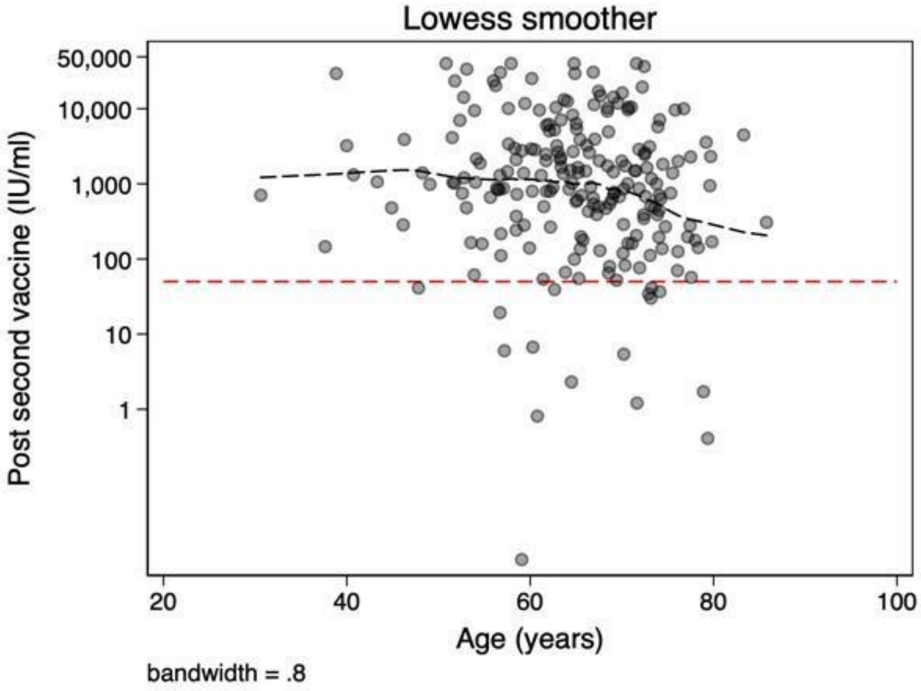

Supplementary Figure 4b. Comparison of Anti-S antibody after second vaccine dose by type of Vaccine

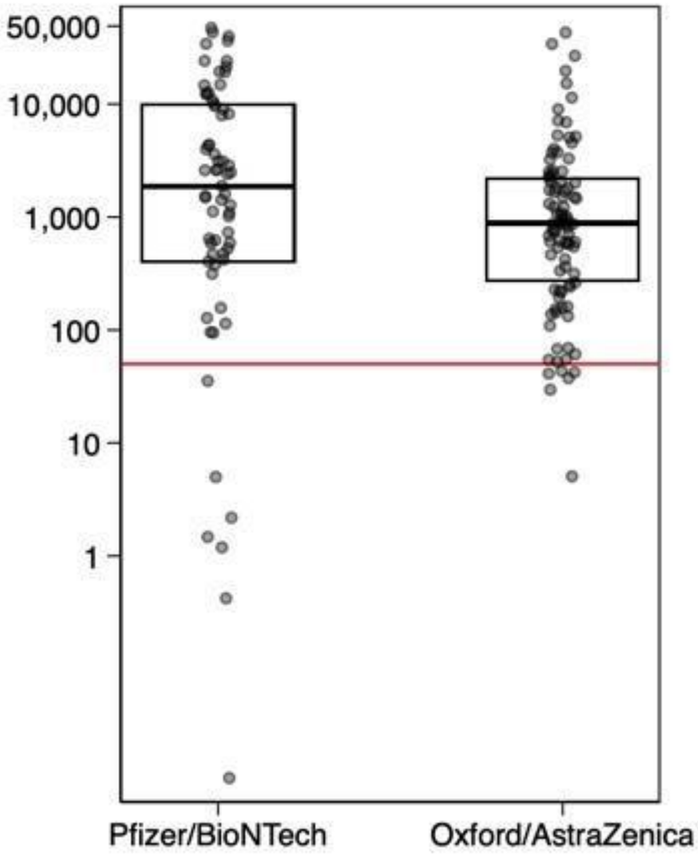

Legend: Comparison of Anti-S values < 50 IU/ml, using Fischer exact test, p=0.39 Kruskal-Wallis p=0.018

Supplementary figure 5a and 5b. Relationship between Anti-S concentration and a) time from second vaccine; b) interval between vaccine doses

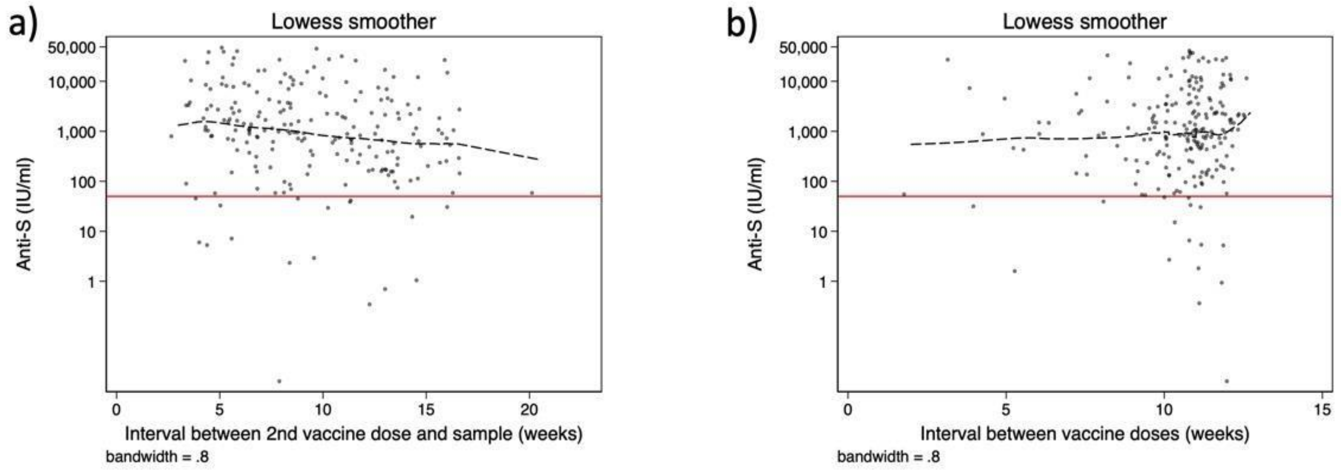

Legend: (a) Dash-lines represents Lowess line with 0.8 bandwidth, Solid red line, threshold of 50 IU/ml. Spearman's rho= -0.12, p=0.002. (b) Dash-lines represents Lowess line with 0.8 bandwidth, Solid red line, threshold of 50 IU/ml. Spearman's rho= 0.085, p=0.22

Supplementary Figure 6. Visual representation of T-spot and humoral responses post vaccination

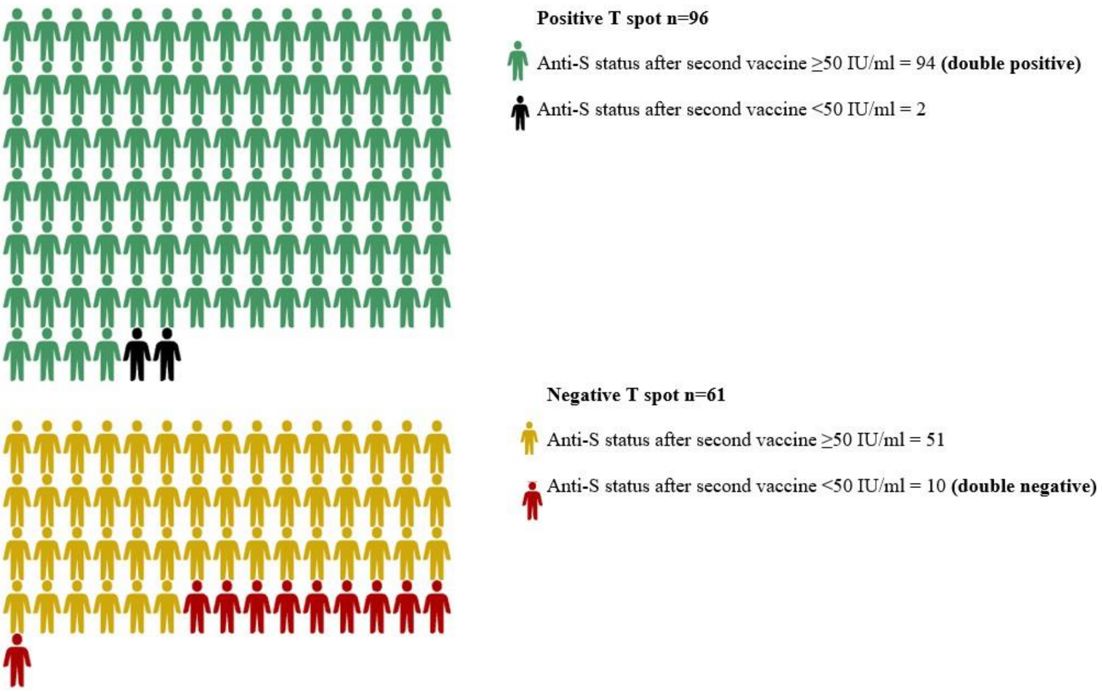

Supplementary Tables

Supplementary Tables and Legends

Supplementary Table 1	Supplementary Table 1. Relationship between IGRA reactivity and Myeloma status and Chemotherapy<br><br>*Legend: CR/VGPR – Complete remission/ Very good partial remission.PR/Stable – Partial remission/ Stable disease. Other chemotherapy included proteasome inhibitors - ixazomib, carfilzomib, bortezomib; immunomodulatory drugs - thalidomide, lenalidomide, pomalidomide; belantamab, bendamustine, cyclophosphamide, dexamethasone, other steroids. bendamustine, cyclophosphamide, dexamethasone, other steroids*

Supplementary Table 1. Relationship between IGRA reactivity and Myeloma status and Chemotherapy

		Myeloma status (p=0.003)				Chemotherapy type (p=0.24)		
		CR/VGPR	PR/Stable	Progressive / Relapse		No therapy	CD38/BCMA	Other
T spot		n=76	n=42	n=22		n=34	n=24	n=36
Negative	n=55	20 (26.3%)	23 (54.8%)	12 (54.6%)	n=37	12 (35.3%)	13 (54.2%)	12 (33.3%)
Positive	n=85	56 (73.7%)	19 (45.2%)	10 (45.5%)	n=57	22 (64.7%)	11 (45.8%)	24 (66.7%)

Legend: CR/VGPR – Complete remission/ Very good partial remission.PR/Stable – Partial remission/ Stable disease. Other chemotherapy included proteasome inhibitors - ixazomib, carfilzomib, bortezomib; immunomodulatory drugs - thalidomide, lenalidomide, pomalidomide; belantamab, bendamustine, cyclophosphamide, dexamethasone, other steroids. bendamustine, cyclophosphamide, dexamethasone, other steroids
